# Vitamin K2 in Health and Disease: A Clinical Perspective

**DOI:** 10.3390/foods13111646

**Published:** 2024-05-24

**Authors:** Tao Zhang, Christine O’Connor, Helen Sheridan, James W. Barlow

**Affiliations:** 1 School of Food Science & Environmental Health, Technological University Dublin, Grangegorman, 7, D07 ADY7 Dublin, Ireland; christine.oconnor@tudublin.ie; 2 The Trinity Centre for Natural Products Research (NatPro), School of Pharmacy and Pharmaceutical Sciences, Trinity College Dublin, 2, D02 PN40 Dublin, Ireland; hsheridn@tcd.ie; 3 School of Pharmacy and Pharmaceutical Sciences, Trinity College Dublin, 2, D02 PN40 Dublin, Ireland; 4 Department of Chemistry, RCSI University of Medicine and Health Sciences, 2, D02 YN77 Dublin, Ireland

**Keywords:** vitamin K2, VK2, menaquinone, health, therapeutic potential

## Abstract

Vitamins are essential organic compounds that vary widely in chemical structure and are vital in small quantities for numerous biochemical and biological functions. They are critical for metabolism, growth, development and maintaining overall health. Vitamins are categorised into two groups: hydrophilic and lipophilic. Vitamin K (VK), a lipophilic vitamin, occurs naturally in two primary forms: phylloquinone (VK1), found in green leafy vegetables and algae, and Menaquinones (VK2), present in certain fermented and animal foods and widely formulated in VK supplements. This review explores the possible factors contributing to VK deficiency, including dietary influences, and discusses the pharmacological and therapeutic potential of supplementary VK2, examining recent global clinical studies on its role in treating diseases such as osteoporosis, osteoarthritis, rheumatoid arthritis, cardiovascular disease, chronic kidney disease, diabetes, neurodegenerative disorders and cancers. The analysis includes a review of published articles from multiple databases, including Scopus, PubMed, Google Scholar, ISI Web of Science and CNKI, focusing on human studies. The findings indicate that VK2 is a versatile vitamin essential for human health and that a broadly positive correlation exists between VK2 supplementation and improved health outcomes. However, clinical data are somewhat inconsistent, highlighting the need for further detailed research into VK2′s metabolic processes, biomarker validation, dose–response relationships, bioavailability and safety. Establishing a Recommended Daily Intake for VK2 could significantly enhance global health.

## 1. Introduction

**Figure 1:**
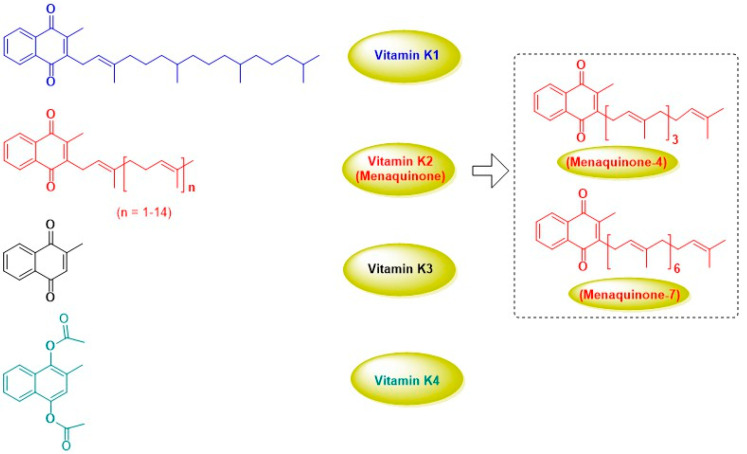
Chemical structures of compounds in the vitamin K family.

Vitamin K (VK) comprises a group of menadione derivatives, first isolated from natural (plant) sources. The key role of vitamin K in blood clotting was first recognized in 1936 by Henrik Dam [1], who observed significant coagulation dysfunction in chickens fed a low-fat diet, resulting in severe bleeding. The later discovery of structurally analogous compounds in fermented foods led to the original vitamin K substance being termed phylloquinone or vitamin K1 (VK1), while those forms contained in some fermented and animal foods were collectively termed menaquinones or vitamin K2 (VK2). VK2 can also be generated by Gram-positive and Gram-negative bacteria in the human body [2]. Vitamin K3 (menadione) and vitamin K4 (menadione acetate), prepared by chemical synthesis, also have pro-coagulative effects. Figure 1 shows the structures of these substances, highlighting their shared bicyclic core. VK1 and VK2 feature a variable side chain at the 3-position of the quinone system: VK1 has a branched aliphatic side chain, featuring one double bond, while VK2 possesses a polymeric side chain with variable numbers of isoprene units [3]. The saturated alkane moiety of VK1 permits free rotation, while the repeating alkene units within VK2 confer strong rigidity and distinct *cis*- and *trans*-isomers, only the *trans* forms of which are biologically active [4]. As polymerised isoprene is of terpenoid origin, from a side chain perspective, VK2 may be considered a terpene, with MK-4 (4 isoprene units) and MK-7 (7 isoprene units).

**Figure 2:**
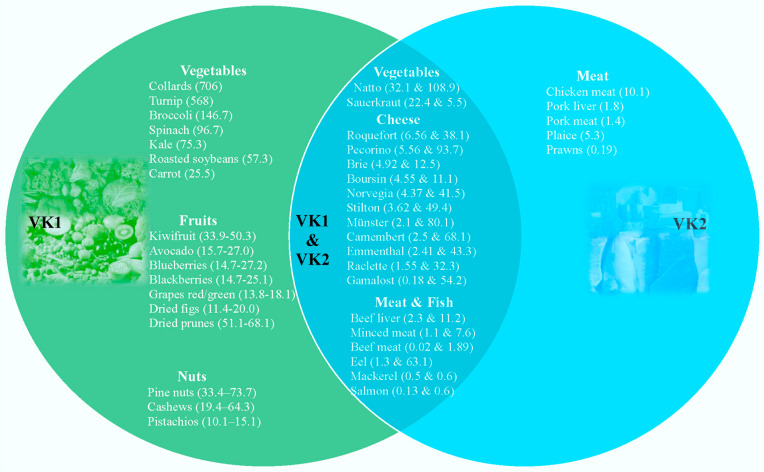
Levels (µg per 100 g of product) of vitamin K1 (VK1) and vitamin K2 (VK2) in different foodstuffs.

VK1 is the main form of VK in the human diet, found mainly in green leafy vegetables (e.g., spinach, cabbage, kale), fruits (e.g., avocado, kiwi, grapes), as well as in some plant oils (e.g., soybean oil) [3]. VK2 is mainly synthesised by gut bacteria and exists in meat, dairy and fermented foods [5,6,7]. Natto, a traditional Japanese food, is noteworthy as the richest known source of VK2, with more than 100 times the VK2 content of other sources. Natto is produced through the fermentation of soybeans with *Bacillus subtilis*, primarily as MK-7 [3]. Dairy products are also rich in VK2, with hard cheeses a notable source [8]. The relevant levels of VK1 and VK2 in foods are shown in Figure 2 [9]. In addition, VK2 (as MK-4) can be synthesised from VK1 in certain animal tissues. This transformation is mediated via the UbiA prenyltransferase domain-containing 1 enzyme and involves menadione as an intermediate [10]. MK-4 derived from ingested VK1 is present at high levels in animals [11], suggesting that VK1 absorbed by herbivores from green leaves is converted into VK2 in the body and can then perform physiological functions that VK1 cannot achieve. This is of great significance in the context of species survival and evolution. It also suggests that humans, on the apex of the food chain, may need a balanced intake of animal and plant foods, which is of significance in considerations of supplementing VK1 and VK2 necessary to maintain homeostasis. In the same vein, vegetarians should consider consuming fermented foodstuffs to maintain this balance. In our previous review on VK2 [12], we demonstrated that VK2 possesses multiple biological functions through the lens of in vivo and in vitro studies and plays a wide range of roles in maintaining human health. In the present study, we reviewed different possible causes of VK2 deficiency and highlighted the clinical evidence for the beneficial roles of VK2 in diverse disease states.

The methodology used to construct this article included a review of published reports from various databases (from inception to 30 November 2022 and updated on 04 December 2023, without language restrictions): Scopus, PubMed, Google Scholar, ISI Web of Science and CNKI, to maximise the retrieval of relevant results. We considered both review and original research articles involving human studies. The search keywords utilised for the literature screening were “vitamin K2” or “menaquinone” in combination with “health” or “diseases”. The database search was supplemented by consulting the bibliography of the articles, reviews and published meta-analyses. The literature research was not limited to a time period, but a particular focus was given to studies from the past 20 years. Relevant articles were chosen after reviewing all titles and abstracts, and full texts were obtained if the information contained in the title or abstract was insufficient to exclude the study. When the form of VK used (whether VK1 or VK2) was not specified, those studies were excluded, unless the outcomes of the studies were considered to be important and significant to report.

## 2. Etiology of Decreased Vitamin K2 Synthesis and Absorption

At present, population-based epidemiological studies of VK2 deficiency are limited, as low levels of VK2 are typically found in normal blood, in the absence of VK2 supplementation or in cases of high intake of VK2-rich foods. There is also no harmonised detection index or cut-off value for evaluating VK2 status (measurement of coagulation factors or prothrombin time is a common surrogate in clinical practice). There is also no distinction between VK1 and VK2 in countries with established dietary recommendations for VK [9]. Despite these observations, it has been noted that VK2 intake from the food sources of today is generally lacking, especially in developing countries [13]. Possible stressors include environmental pollution, industrial farming practices, artificial feeding, residues of pesticides and fertilisers in agricultural products and the widespread use of preservatives, additives and antibiotics. All these could have an impact on VK2 intake and the synthesis of endogenous VK2. Eating foods rich in VK2 could be a good option to counter declining levels, but in practice, many people, due to dietary preferences (certain Asian diets, vegans, or vegetarians) may struggle to achieve this. Further possible causes of VK2 deficiency are an imbalance in intestinal flora, issues regarding in vivo absorption and/or metabolism and drug–drug interactions.

### 2.1. The Imbalance of Intestinal Flora

Although the intestinal microbiome can produce a small amount of endogenous VK2, these levels may not meet the needs of the human body, and, in addition, colonic absorption of VK2 is limited [14]. Modernisation of agricultural production, with resulting residues of pesticides and fertilizers in agricultural products and the practices of the food processing industry, including the use of preservatives and artificial food additives, have caused the dysregulation of human intestinal flora [15,16], further impacting negatively upon VK2 production. Menaquinones have been highlighted as a major class of growth factors for taxonomically diverse bacteria from the human gut microbiome, including *Faecalibacterium*, *Bacteroides*, *Bilophila*, *Gordonibacter* and *Sutterella* species [17]. The importance of food quality and a well-balanced diet, particularly with ageing, has been highlighted in a recent review of vitamin K in the context of diet and the gut microbiome [18]. An interesting observation is the fact that VK1 absorption from fruit and vegetables may be lower than that from processed plant oils associated with unhealthy diets; likewise, for VK2, processed meat products are a significant source of menaquinones, alongside more healthy choices such as fermented foods. Also, exogenous VK may undergo microbiome-initiated remodelling [19]. The complexities of the impact of our dietary choices on both VK bioavailability and modulating gut microbiome composition, and therefore VK status, are increasingly realised.

Antibiotics can also reduce VK2 production by destroying VK2-producing bacteria in the gut. This effect is particularly pronounced with cephalosporins such as cefoperazone [20]. In addition, proton pump inhibitors can also impact VK2 status [21], possibly through effects on the intestinal flora.

### 2.2. Physiological and Genetic Factors

Many objective physiological factors influence VK2 absorption and utilisation. The absorptive capacity of the human gastrointestinal tract gradually declines with age, which could cause VK2 deficiency in middle-aged and elderly people and, thus, merit dietary supplementation [22]. In addition, the decreased absorptive ability of newborns, patients with liver and gallbladder diseases and elderly patients with metabolic diseases could also lead to VK2 deficiency [22,23].

Experimental evidence in this regard was shown by Holden and colleagues [24]. In this observational and prospective study involving 167 chronic kidney disease (CKD) patients, it was observed that patients with the CG/GG genotype of vitamin K epoxide reductase complex subunit 1 (the target enzyme for warfarin) had a higher risk of coronary artery calcification (CAC) progression and poorer survival, suggesting the potential role of VK2.

Gamma-glutamyl carboxylase (GGCX) gene single nucleotide polymorphism (R325Q, 974G > A) is associated with bone mineral density (BMD). Haraikawa et al. studied the effect of GGCX gene single nucleotide polymorphism (974G > A) on vitamin K intake, serum vitamin K levels and the ratio of uncarboxylated osteocalcin (ucOC) to carboxylated osteocalcin (OC) in healthy young Japanese subjects. The results showed a significant negative correlation (*p* < 0.001) between the ucOC/OC ratio and vitamin K intake in homozygotes (GG type) and heterozygotes (GA type) [25]. Therefore, appropriate supplementation of vitamin K2 is warranted for people with high-risk genotypes (GG or GA).

### 2.3. Drug Interactions

Vitamin K interacts with certain drugs, which affect the concentration and action of VK2 in the body. Warfarin is a coumarin anticoagulant and vitamin K antagonist (VKA), blocking the circulation of VK in the liver and periphery, leading to secondary vitamin K deficiency, which has a negative impact on blood vessels, bones, kidneys, brain and other tissues and systems (e.g., inflammation, immune function and tumours). VK2 has the potential for serious drug interactions with other coumarin anticoagulants, such as phenprocoumon, acenocoumarol and tioclomarol [26].

The cholesterol-lowering drugs cholestyramine and colestipol are anion-exchange resins that reduce cholesterol levels by preventing the reabsorption of bile acids, but they also decrease the absorption of VK and other lipid-soluble vitamins [27,28]. Orlistat is a weight loss drug that reduces intestinal absorption of dietary fat but may also reduce VK absorption [29]. However, in a study of obese teenagers taking supplementary vitamins, including 25 µg VK (as synthetic VK1, phytonadione), there was a nonsignificant decrease in VK status [29].

Statins are commonly used lipid-lowering drugs, lowering cholesterol and thereby reducing atherosclerosis. However, it has been shown that statins may cause CAC. A possible mechanism may be a toxic effect on mitochondria, impairing muscle function in the heart and blood vessels by depleting Coenzyme Q10 and heme A. Statins also inhibit VK2 synthesis [30]. VK2 is a cofactor for the activation of matrix Gla protein (MGP), which protects arteries from calcification. It has been postulated that the high incidence of heart failure and atherosclerosis may thus be paradoxically exacerbated by the widespread use of statins [28,31].

Patients with CKD often experience hyperphosphatemia, and treatment with phosphorus-lowering drugs is required. Studies have shown that phosphate binders have adverse effects on the bioavailability of VK2. In vitro experiments have shown that calcium acetate/magnesium carbonate, lanthanum carbonate, calcium carbonate and sevelamer carbonate can all combine with VK2, which affects the absorption of VK2 and may counteract the ability of VK2 to inhibit vascular calcification [32]. In a study of various CKD patients taking phosphate binders, dephosphorylated-uncarboxylated MGP levels were higher in those taking sevelamer, suggesting reduced vitamin K status [33]. However, the clinical relevance of these interactions has been challenged [34].

## 3. Potential Therapeutic Benefits

**Figure 3:**
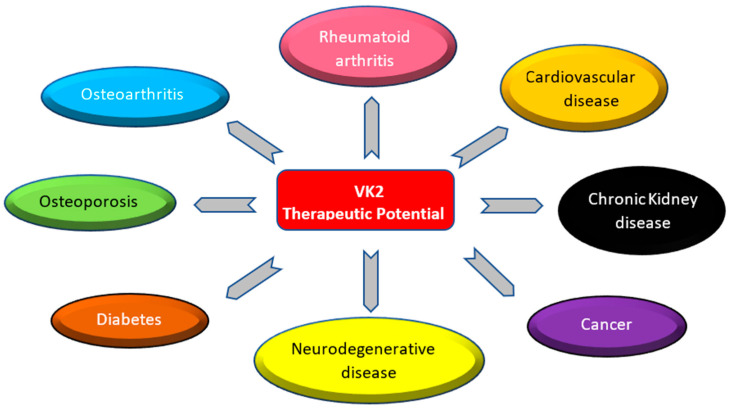
The therapeutic potential of VK2 in various disease states.

Research conducted in recent decades has suggested the protective roles of VK2 in tissue mineralisation-, inflammation-, oxidation- and age-related conditions, which could provide new directions for VK2 in future clinical practice. The aim of this section is to summarise and analyse recent available clinical evidence on the relationship between VK2 intake and status and to describe its modulatory effects in various disease states (Figure 3).

### 3.1. Osteoporosis

Osteoporosis is a metabolic skeletal disorder, characterised by decreased BMD, fragile bone and increased risk of fracture. BMD typically declines with age, particularly in postmenopausal women [35], and has important consequences on general health and quality of life. Many factors are associated with osteoporosis, including genetics, age, sex, ethnicity and hormone levels, in addition to lifestyle variables such as smoking, diet and alcohol [36]. An estimated nine million fractures annually are caused by osteoporosis [37].

Long-established preventative interventions such as calcium supplementation have been challenged, due to a claimed association with an increased risk of heart disease through enhanced calcification of blood vessel walls and soft tissue [38]. More evidence-based preventative strategies are thus needed. The role of VK2 in both diet and in supplementary form has been evaluated. In Japan, researchers established a positive relationship between intake of the fermented soybean product, natto (containing VK2 380 μg/pack and VK1 20 μg/pack), and BMD among 1662 healthy men [39]. Various studies of VK, typically co-administered with vitamin D, at typical VK2 doses ranging up to 45 mg/d, have shown increases in BMD and a reduced incidence of fractures [40].

In a meta-analysis conducted by Fang et al., VK supplementation (VK1, VK2) effectively increased BMD at the lumbar spine, but not the femoral neck. The weighted mean difference in absolute BMD change was 21.60 mg/cm^2^ at the lumbar spine, but only 0.25 mg/cm^2^ at the femoral neck, while the relative change was 1.27% at the lumbar spine and 0.17% at the femoral neck. A subgroup analysis revealed a significantly favourable effect on lumbar spine BMD by VK2, rather than VK1. Moreover, ethnicity, gender and VK type were also associated with variable effects on BMD in the lumbar spine. Most of the included trials were conducted in women only [41]. In a more recently published review on VK2 and other nutrients and bone health [42], different effects of VK2 on BMD and fracture risk from several meta-analyses were reported [43,44], with many seeming to confirm the potential benefits of VK2 supplementation in osteoporotic patients, possibly via enhancement of the effects of calcium and vitamin D. However, biases in several studies were noted. As a result, the authors suggested that the efficacy of VK intake and/or supplementation to prevent or treat sarcopenia needs to be further studied, due to insufficient data [42]. Similar findings were echoed in an even more recent meta-analysis [45]. More specifically, the efficiency of MK-7 supplementation was shown, as it accumulates to 7–8-fold higher levels than VK1 upon chronic administration and significantly promotes γ-carboxylation of OC, increasing cOC levels while decreasing ucOC plasma levels, thus promoting bone metabolism [46,47]. Inaba et al. further suggested that >100 µg/d of MK-7 supplementation could be considered for enhancing bone health [46].

The synergistic effect of VK2 supplementation with antiresorptives has also been explored. Co-administration with alendronate facilitated the decrease in bone turnover markers and ucOC levels in post-menopausal women [48] and, with anabolic agents (teriparatide), increased osteoblastic lineage activation and bone healing in animals [49].

Finally, a body of evidence, from in vitro and in vivo studies, supports the pathogenic role of oxidative stress contributing to osteoporosis (especially in postmenopausal osteoporosis), most likely in synergy with inflammation [50]. Therefore, employing VK supplements including VK2 as dietary antioxidants, with the aim of decreasing levels of oxidative stress with possible beneficial effects on bone, has been suggested [3].

In Japan, VK2 is recommended as a second-line drug in guidelines on the management and treatment of glucocorticoid-induced osteoporosis by the Japanese Society for Bone and Mineral Research [51], and a 250–300 µg of daily intake of MK-7 is also recommended as a therapeutic and prophylactic medication for osteoporosis [52]. In Europe, the adequate daily intake of VK from food (as VK1) was set at 70 μg by The European Food Safety Authority for all adults, including pregnant and lactating women [53].

### 3.2. Osteoarthritis (OA) and Rheumatoid Arthritis (RA)

Currently, there are no effective treatments to treat OA pathogenesis, and available clinical data regarding the effects of VK on OA are also limited in providing definitive cause–effect information [22]. Certain vitamin K-dependent proteins are found in joint tissues, including cartilage, bone and periosteum, and imbalance in the action of these proteins due to genetic factors is associated with OA [54]. Evidence from both animal and human studies suggests VK status may be associated with OA risk. Specifically, a population-based cross-sectional study of 719 Japanese participants (449 females, ≥60 years) showed that an increased risk of knee OA development was associated with lower VK intake [55]. This finding was in line with the prior Framingham Offspring study, which involved 672 participants (358 females; mean age of 65.6). After adjustment for age, sex, body mass index, total energy intake, plasma vitamin D and femoral neck BMD, the data supported an inverse association of VK status with OA in the hand and knee [56]. Properly designed clinical trials of VK supplementation in OA patients are thus warranted.

Some evidence exists demonstrating a modulating effect of VK2 in inflammation-based arthritis, such as rheumatoid arthritis (RA). Ebina et al. presented both cross-sectional and longitudinal associations between VK2 supplementation (45 mg/d) and significantly reduced inflammation in RA patients [measured by marker proteins including C-reactive protein (CRP)], as compared to the VK2-naïve group [57]. In an in vitro study using mitogen-activated peripheral blood mononuclear cells of healthy subjects and RA patients, significantly enhanced immunosuppressive efficacy of methotrexate by VK2 was demonstrated [58]. Similarly, another cross-sectional study also showed that RA patients who received 100 μg/d of MK-7 for 3 months showed a significant decrease in disease activity score, along with improved biomarker levels [erythrocyte sedimentation rate (ESR), CRP and MMP-3] [59]. Human studies on MK-4 in RA patients are still lacking, but several in vitro and in vivo studies have supported the anti-inflammatory action of MK-4 [60,61].

### 3.3. Cardiovascular Disease (CVD)

Cardiovascular calcification, mediated via various pathophysiological processes, is a central contributor to CVDs. MGP deficiency and inactivation of MGP has been repeatedly associated with VK deficiency, resulting in stimulation of all types of calcification and contributing to the development of cardiovascular events, whereas VK status (especially VK2) appears to inversely correlate with CVD risk [62]. With the aim of examining the relationship between dietary VK intake and the incidence of coronary heart disease (CHD), Gast et al. utilised data from the Prospect—The European Prevalence of Infection in Intensive Care (EPIC) study in the Netherlands. In this study, a cohort of 16,057 women between 49 and 70 years with no previous history of CVD was followed for 8.1 years [63]. The results indicated a significant negative relationship between a high VK2 intake (especially MK-7, MK-8, MK-9) and a reduced risk of CHD, after adjustment for known risk and dietary factors. Similar results were also obtained in the Rotterdam study, following 4807 participants free from myocardial infarction at baseline over a 7-year period [64]. In both these studies, VK1 intake did not significantly impact on the outcomes. However, in a more recent prospective cohort study involving 53,372 participants with 17–22 years of follow-up, both dietary VK1 and VK2 intakes were inversely related to atherosclerotic CVD hospitalisation risk [65].

CVD risk is strongly associated with kidney dysfunction, due to enhanced vascular calcification in these patients, with even the early stages of CKD causing hypertension and potentiation of the risk of CVD [66]. More than 50% of patients undergoing dialysis develop CVD, and mortality in haemodialysis patients due to CVD is 20 times higher than in the general population [67]. Intimal atherosclerotic plaque calcification and medial calcification are commonly observed in these patients [68].

To evaluate the influence of VK supplementation on the development of cardiovascular calcification in the context of CVD, several randomised and placebo-controlled trials are currently ongoing or recently completed. The Valkyrie trial investigated the effects of VK2 supplementation alongside rivaroxaban in place of VKAs on the progression of vascular calcification among haemodialysis patients (NCT02610933), and the outcomes are pending [69]. However, in a separate clinical study, VK status improved significantly upon withdrawal of VKAs and thrice-weekly supplementation of VK2 (2 mg), changes in coronary artery, thoracic aorta and cardiac valve calcium scores and pulse wave velocity were not different among the treatment arms [70]. Importantly, the intervention did not normalise systemic dephosphorylated-uncarboxylated (dp-uc)MGP levels among patients. In another study (EudraCT Number: 2019-004906-88 and NTR number: NL7687) investigating the effect of MK-7 supplementation on serum calcification propensity and arterial stiffness in vitamin K-deficient kidney transplant recipients, supplementation did not alter serum calcification propensity but prevented progression of arterial stiffness, suggesting that vitamin K has vascular effects independent of calciprotein particles [71]. In a diabetic cohort with kidney disease, MK-7 supplementation (375 µg/d, 24 weeks) lowered the rate of progression of arterial stiffness in chronic haemodialysis patients, in an open-label, multicentre randomised controlled study (TCTR20230217001) [72], while another study of arterial calcification among diabetic patients showed that six months’ supplementation with VK (as 360 μg MK-7) did not improve serum calcification propensity time [73].

Two years’ supplementation with MK-7 (720 µg/d) alongside vitamin D (25 µg/d) did not reduce the progression rate of aortic valve calcification in patients with aortic stenosis, in a randomised, double-blind and multicentre trial study (NCT03243890) [74]. The BASIK2 trial of bicuspid aortic valve stenosis is evaluating the effect of VK2 on calcification using ^18^F-sodium fluoride positron emission tomography/magnetic resonance (NCT02917525) [75].The VitaK-CAC trial is evaluating MK-7 supplementation to reduce vascular calcification in patients with coronary artery disease (NCT01002157) [76], while a further trial is anticipated to start in 2024 to study the effect of VK2 supplementation on arterial micro-calcification as assessed by PET/MRI in Carotid Artery Disease (INTRICATE) (NCT04010578) [77]. The InterVitamin K trial (NCT05259046) is currently investigating the effect of daily MK-7 supplementation over three years on the progression of vascular calcification in terms of the CAC score in individuals with detectable vascular calcification [78].

### 3.4. Chronic Kidney Disease (CKD)

Further to the association between CKD-related calcification and CVD already discussed in Section 3.3, VK deficiency is seen across patients with diverse CKDs, including urolithiasis, nephrolithiasis and uraemia [79]. VK status becomes more significant with progression through CKD stages [80] and when undergoing maintenance haemodialysis [81]. It has been shown in both animal models and human studies that MGP is overexpressed as renal dysfunction progresses and can thus indicate deterioration of renal function [82].

A multivariate analysis involving 107 patients in all five stages of CKD showed that dp-ucMGP status was a predictor of arterial calcification, independent of age, gender, previous CVD and high total-ucMGP levels, progressively augmented with the CKD stage and positively correlated with aortic calcification [83]. In a study of 141 CKD stage-5 patients, Jaminon et al. demonstrated that plasma levels of circulating dp-ucMGP were an independent predictor of increased vascular calcification in patients with end-stage CKD and correlated with both higher coronary artery calcium scores and degree of medial calcification [84]. Moreover, etiologic in vivo evidence has shown that vascular VK deficiency in uraemia may partially result from a decrease in γ-carboxylase activity, leading to an increase in uremic vascular calcification [85] and the exposure of the vasculature to a toxic uremic milieu [86].

It has further been suggested that VK intake could contribute to CKD treatment by modulating MGP, since MGP is abundantly expressed in the kidney, 5-fold more so than in bone [87]. The effect of high-dose (100 µg/g diet) MK-4 supplementation on restoration of MGP functionality in a murine CKD model has been demonstrated, suggesting that MK-4 may offer a protective effect against cardiovascular calcification in this situation [85].

In a trial involving 50 haemodialysis patients, the average decrease in plasma dp-ucMGP levels at week 4 following MK-7 treatment (360 μg/d) was found to be 86%, with the lowest drop rate (*p* = 0.01) in diabetics [88]. Caluwe et al. emphasised the importance of dose and time dependency of VK2 supplementation in the carboxylation of MGP in a randomised dose-finding study of 200 haemodialysis patients. MK-7 supplementation dose-dependently reduced dp-ucMGP levels by 17, 33 and 46% after receiving 360, 720 or 1080 μg of MK-7 treatment (thrice weekly for 2 months), respectively [68]. Delanaye and coauthors showed a rapid time-effect decrease in serum dp-ucMGP levels (by at least 40%) within 5 days after terminating VK antagonist treatment in seven stable haemodialysis patients [89]. Moreover, in a 270-day trial with 38 non-dialysis CKD patients, supplementation with combined MK-7 and vitamin D resulted in a decrease in plasma dp-ucMGP level by 10.7%, whereas supplementation with vitamin D alone showed no change [90]. Nigwekar et al. showed that every 0.1-unit reduction in plasma cMGP level was associated with a more than two-fold increase in calciphylaxis risk in haemodialysis patients, suggesting that aberrant VK-mediated MGP carboxylation may have a role in the pathogenesis of calciphylaxis [91]. The use of MK-7 as the VK2 form of choice in these trials probably reflects its longer half-life (72 h) than that of MK-4 (1 h).

Additionally, Wei et al. confirmed the inverse relationship between decreased estimated glomerular filtration rate (eGFR) and risk of renal impairment with higher dp-ucMGP levels in a study involving 1166 white Flemish patients (mean age 38.2 years) and 714 South Africans (49.2% Black; 40.6 years) [92], further indicating the use of dp-ucMGP as a biomarker predicting deterioration of renal function in the general population, assessed by a decrease in eGFR and increase in albuminuria [93]. This further suggests that VK supplementation to modulate MGP not only results in inhibition of arterial calcification but also protects renal function. Despite all these observations, supplementary VK2 does not appear to translate into unambiguous cardiovascular protection in chronic CKD patients. In a trial of 159 patients (mean age 66) with CKD, VK2 supplementation did not improve vascular stiffness or other measures of vascular health [94]. Likewise, in a meta-analysis of ten trials, while both levels of relevant biomarkers of vascular calcification and elasticity improved, significant changes in calcification scores were not observed [95].

### 3.5. Diabetes and the Metabolic Syndrome

Studies conducted over the past two decades have lent additional support to the role of VK in diabetes, mainly Type 2-diabetes (T2-D). Dietary/supplementary VK2 intake has been associated with significant changes in insulin concentration and sensitivity in human studies, mostly focused on MK-4 and MK-7, administered at mg and µg dose levels, respectively, due to the observed difference in their bioavailability [96]. Choi et al. demonstrated that high MK-4 intake (30 mg/d) contributed to a significant increase in insulin sensitivity index in healthy young men (*n* = 18, 25.5–31.5 years), compared to the placebo group (*n* = 15, 24–31 years) over 4 weeks [97]. Similarly, a linear, inverse association between VK intake (average intakes: VK1 200 ± 98 μg/d, menaquinones 31 ± 7 μg/d) and a reduced risk of T2-D was also shown in a long and large study conducted by Beulens et al. [98].

Evidence of the beneficial effects of MK-4 and VK1 on glucose metabolism/homeostasis has also been provided by streptozotocin (STZ)-induced Type 1 diabetic rat models [99], while Khalil et al. demonstrated the protective function of MK-7 against impaired glucose homeostasis in ovariectomised exercised and nonexercised rats [100], with decreases in glucose levels and increases in insulin, lipocalcin-2 and adiponectin levels shown in both models. Similar findings were also observed in another rat study, whereby MK-4 and VK1 (600 mg/kg diet, 3-month) treatment significantly reduced fat accumulation and serum triglycerides by 48% (MK-4) and 29% (VK1), compared to the control group [101], suggesting a potential role of VK, especially VK2, in treatment of IR via its lipid-lowering effects [96].

The beneficial synergistic effects of vitamins D, K (90 µg MK-7) and Ca co-supplementation on maximum levels of left carotid intima-media thickness and metabolic status in T2-D patients with CHD was demonstrated by Asemi et al. over 12 weeks in a randomised, double-blind, placebo-controlled trial. Significant changes in insulin levels, homeostasis model assessment for insulin resistance (HOMA-IR) and HOMA-β-cell function (HOMB-B) were noted [102]. Conversely, a randomised, double-blind, placebo-controlled trial involving 68 T2-D patients (aged 30–70) did not show significant improvement in various IR-related indices following 12-week MK-7 intake (360 μg/d) [103]. In a recent case–control study (NCT04387019) involving 60 T2-D patients (30 each in controlled and uncontrolled groups) and 30 non-diabetic controls, serum VK2 level was found to be significantly lower, especially with uncontrolled hyperglycaemia, than in the control group. This indicates the possible relationship between VK2 level and glycaemic homeostasis in T2-D patients [104]. The clinical implementation of VK2 intervention for diabetes management was further suggested by another 6–month clinical trial (ChiCTR1800019663) with 60 T2-D participants and MK-7 intervention [105].

The connection between VK2 and the metabolic syndrome (MetS) has also been explored. MetS represents a combination of risk factors which increase the risk of development of diabetes and CVDs and includes abdominal obesity (waist circumference), moderate hypertension, low high-density lipoprotein (HDL) cholesterol, high glucose level and high triacylglycerol concentrations [106]. In a 10-year follow-up study including women (*n* = 402; 49–70 years) and men (*n* = 400; 40–80 years), Dam et al. showed cross-sectionally and longitudinally that high VK2 intake (210.3 ± 127.0 μg/d) was associated with a decreased occurrence of MetS (measured as lower triacylglycerol levels and waist circumference), but interestingly, no such correlation was observed in the VK1 treatment group (31.1 ± 12.5 μg/d) [107]. Conversely, a cross-sectional study with a 6-year follow-up involving 5800 adults (20–45 years) suggested that higher VK1 intake (84.8 ± 3.2 μg/d) may favourably influence the prevalence of MetS (measured as decreased high blood pressure and reduced HDL cholesterol) [108].

In the context of molecular mechanisms, studies provide insight into the involvement of OC in pancreatic β-cell proliferation, insulin expression, adiponectin expression in adipocytes, as well as glucose metabolism, suggesting beneficial effects of VK in diabetes via OC metabolism [96,109]. The association of higher cOC after VK2 intake with improved insulin sensitivity [97], lowered IR [110], decreased body mass and BMI [111] has repeatedly been established by human studies. However, other inconsistent results have been reported. Lee et al. reported a modulatory role of ucOC in regulating glucose metabolism and increasing β-cell proliferation and insulin secretion in a genetically modified mice model [112]. Iki et al. reported an inverse relationship between serum undercarboxylated OC levels and glycaemic status/IR in their study involving 2174 Japanese men [113]. Furthermore, the effect of VK in improving insulin sensitivity via inhibiting anti-inflammatory responses, through inactivation of NFκB signalling pathway and suppressions of IL-6, IL-1β, TNF-α expression, has also been reported [61,96]. More recently, SIRT1 signalling pathway in upmodulating mitochondrial function to protect against IR has been demonstrated [114].

### 3.6. Neurodegenerative Diseases

Neurodegenerative disorders comprise a wide spectrum of neurological disorders with variable clinical phenotypes, including behavioural and cognitive changes, and affect millions of people worldwide. Of these disorders, Alzheimer’s disease (AD) and Parkinson’s disease [86] are the two most common neurodegenerative diseases [115]. The risk of being affected by a neurodegenerative disease increases dramatically with age. Therefore, as the population ages, estimates of such diseases increase in parallel, with dementia expected to affect 81 million people by 2040 [115,116]. VK may serve to support neurological function through several mechanisms. The dependence of sphingolipid synthesis, and therefore myelination of neurons, on VK is well established. Suggested mechanisms of action of VK in preventing cognitive decline, and specifically AD, include inhibition of apoptotic signalling proteins and oxidative stress, including inhibition of p38 MAP kinase [117]. In our previous review, we discussed the potential pathways of the protective effect of VK2 on neuroprotection through modulating neurodegeneration, inflammation and oxidative stress with or without the involvement of VK-dependent proteins (Gas6, Protein S) [12].

The relationship of VK (mainly MK-4 and VK1) with cognitive and behavioural performance is supported by a body of evidence [118]. Studies on the nutritional status of community-dwelling elder patients with early-stage AD revealed that the patient group had significantly lower VK intake than those of age- and sex-matched cognitively intact control participants, even after adjusting for energy intakes [10,119]. On the other hand, in a longitudinal ageing study of 599 middle-aged adults (aged 55–65) with a 6-year follow-up, examining the association between VK status (by dp-ucMGP) and cognitive decline, no direct correlation was found [120]. Fewer studies exist in PD; one small case–control study including 93 PD patients suggested that deficiency of VK2 is associated with PD progression [121].

Some studies have focused on the impact of brain metabolism by VKAs used as anticoagulants (i.e., warfarin, acenocoumarol and fluindione). Ferland et al. demonstrated an association between VKA usage and significantly diminished performances in visual memory and verbal fluency in a large prospective study of 7133 non-demented community dwellers (aged ≥ 65), with a 10-year follow-up. However, no correlation was found between VKA treatment and MMSE scores [122]. In another longitudinal prospective study with a 24-month follow-up involving 378 geriatric outpatients (mean age 82.3 ± 5.6), the authors found those taking VKAs had more severe executive dysfunction (frontal assessment battery) at baseline and incident executive decline over 24 months. Again, no significant association with MMSE scores was seen [123]. The same research group performed another study of brain morphological changes in patients taking VKAs and observed focal atrophies in older adults exposed to VKAs (18 community-dwellers), compared to 36 matched controls [124]. Annweiler et al. further showed that fluindione treatment specifically caused more frequent cognitive impairment among 267 geriatric patients (56.9% female; mean age 83.4 ± 8.1 years) [125].

Trends have been shown between VKA usage and cognitive impairment in patients with atrial fibrillation, regardless of dependency or frailty [126], while the usage of non-VKA-based anticoagulants and a lower risk of cognitive impairment [127] has also been documented. In an attempt to define brain areas affected by VKAs, Tamadon-Nejad et al. used a rat study to show that warfarin-treated rats had a dramatic decrease in MK-4 levels in all brain regions and scored worse than controls in tests of cognition and behaviour, including longer latencies in the MWM test, lower locomotor activity and exploratory behavior, as well as having altered sphingolipid levels, notably those of the ganglioside family. These results suggested that the biosynthetic pathway of MK-4 is suppressed by warfarin, even in the presence of high local VK1 concentrations [128]. In a study involving 85 patients (≥75 years) on VKA treatment, a positive correlation was shown between VK1 concentration and cognitive status [129].

Apart from conditions of impaired cognition, other neurological disorders have been associated with VK. Multiple sclerosis [94] is a complex chronic inflammatory and degenerative disorder of the central nervous system. The immuno-regulatory role of Gas6 has been suggested to be associated with this autoimmune disease via Gas6/TAM systems [130]. In a study conducted by Lasemi et al., substantially lower VK2 levels were detected in MS patients (female: 31 and male: 14) than controls (19 female, 10 male), with VK2 levels in female patients significantly lower than those of males [131]. In an in vivo rat model of anxiety and depression, it was suggested that VK2 might have a regulatory effect on preventing their development, possibly via its effects on blood glucose [132]. Peripheral neuropathy is a frequent and severe complication in people with diabetes. In a study of 198 T2-D patients (mean age 64 ± 8.4), 15.7% of patients with peripheral neuropathy had significantly lower levels of dp-ucMGP, suggesting this fully inactive MGP form could be a biomarker of sensitive neuropathy [133]. The therapeutic effects of MK-7 in relieving peripheral neuropathy symptoms have been shown in a trial of 100 patients (aged 18–65) with peripheral neuropathy (suffering from T2-D or vitamin B12 deficiency), who received 8 weeks of treatment with 200 mg MK-7 daily. The treatment resulted in significant improvements in symptom intensity and was well tolerated by all patients [134]. Surprisingly, one study (ACTRN12615000750583 and ACTRN12617000640303) involving 1347 community-dwelling older women (≥70 years) showed that high VK1 intakes was associated with better physical function and reduced long-term risk of injurious falls, potentially due to improved neuromuscular coordination and vascular function, but no such beneficial relationship was observed with VK2 supplementation [135].

### 3.7. Cancer

The association of VK with cancer can be considered through the lens of chemoprevention, or as a treatment strategy, either alone or as adjuvant chemotherapy. VK has been reported to have an inhibitory role in cancer cell growth, inducing apoptosis and cell-cycle arrest in many cancer lineages, including reproductive, haematological and gastrointestinal lines. Mechanistically, besides the induction of cell-cycle arrest, cell differentiation and apoptosis, the anti-cancer effects of VK2 through autophagy and the suppression of cancer cell invasion have been proposed [136]. In cultured leukaemia cells, VK2 induced autophagy and apoptosis simultaneously [137]. While induced autophagy by VK2 was significant in cholangiocellular carcinoma cells, apoptosis/cell-cycle arrest was inconspicuous [138]. Clinically, accounts of remission in several, diverse cancers have been reported, with translation into improved prognosis in several trials [136,139]. However, the potential of VK as a possible chemopreventive is clouded by contradictory observations.

A significant inverse association between dietary VK2 intake and the risk of prostate and lung cancer incidence was identified in the Heidelberg cohort of the European Prospective Investigation into Cancer and Nutrition study, involving 24,340 participants (aged 35–64). The authors further reported that no such association was observed with VK1 intake [140]. Dasari et al. have demonstrated VK2′s effect on castration-resistant prostate cancer by inducing VCap cell death through specifically targeting ROS-mediated apoptosis and cell-cycle progression as well as metastasis-inhibiting signalling molecules [141]. In breast cancer, while some in vitro studies show promise, such as the demonstration that VK2 can induce non-apoptotic cell death and autophagy in triple negative breast cancer cell lines [142], clinical studies may show conflicting results. For example, a positive relationship between the risk of occurrence and death of breast cancer and total VK (VK1 and VK2) intake was shown in a prospective cohort (US) study involving 51,662 women [143].

Myelodysplastic syndrome (MDS) refers to a category of clonal haematological disorders characterised by dysplastic features of bone marrow cells, ineffective haematopoiesis and cytopenia and carries over 30% risk of progression to acute myeloid leukaemia (AML). A questionnaire survey of multi-centre pilot studies in Japan demonstrated haematological improvement in some MDS/post-MDS AML patients following VK2 (as MK-4, in doses ranging from 20 to 135 mg/day p.o. and 10 to 50 mg/day IV MK-4) administration over 2 years [144]. The efficacy of VK2 treatment was observed in around 30% of patients in these studies, and no adverse effects were noted. These findings correlated with previous case reports, including a female MDS patient (aged 80) treated with MK-4 at 45 mg/d [145] and a female relapsing acute promyelocytic leukaemia patient, who received combination treatment with VK2 (20 mg/d) and all-*trans* retinoic acid [146]. In vitro evidence suggests dichotomous effects of VK2 in leukaemia cells [147] and that it causes suppression of cMYC [148] while improving haematopoiesis and promoting anti-apoptosis of normal erythroid progenitors and differentiation of myeloid progenitors [149]. The pro-apoptotic effects of co-culture of bone marrow mesenchymal stromal/stem cells (BM-MSCs) with MDS-derived cells were enhanced by MK-4 [150]. VK2 may thus offer potential as an adjunctive anti-cancer therapy in this cohort, especially for elderly patients who cannot tolerate intensive chemotherapy and stem cell transplantation.

Other mechanisms of VK2-induced leukemic cell apoptosis and differentiation include upregulation of p27 protein expression [147], disruption of mitochondrial membrane potential by VK2-Bcl antagonist killer 1 (Bak) binding [151], cleavage of PARP and down-regulation of cyclin A2 expression [152]. VK2-initiated mitochondrial apoptosis and ROS generation pathways were also observed in cultured studies of myeloma cells [153] and human ovarian cancer TYK-nu cells [154]. Promotion of HL-60 leukaemia cell differentiation was shown with a cotylenin A/VK2 combination [148], while VK2 promoted greater induction of monocytic differentiation as compared with that by vitamin D3 alone in leukaemia cells [155].

VK2 (as MK-4) appeared to have a positive effect in preventing the development of hepatocellular carcinoma (HCC), the third leading cause of cancer mortality, in patients with type-C cirrhosis, when administered at a dose of 45 mg/d [156]. A suppressive effect of VK2 on HCC recurrence rate and a beneficial effect on survival [157], as well as HCC recurrence derived from hepatitis C viral infection [158], has also been reported, but the results from these studies were not statistically significant. In a prospective randomised controlled trial of 101 HCC post-hepatectomy patients, MK-4 treatment (45 mg/d) exhibited a moderately suppressive effect on HCC recurrence [159]. Conversely, in another double-blind, randomised, placebo-controlled trial of 548 patients, VK2, at either 45 or 90 mg/d, did not prevent HCC recurrence or death [160]. Potential limitations associated with this study, as suggested in a later review, included patient recruitment and MK-4 quality [136]. Additionally, an interactive effect of VK2 combined with vitamin E treatment was suggested by a case study of a male HCC patient (age 65), whereby observation of tumour growth suppression and intraperitoneal dissemination disappearance was noted [161]. A combination of VK2 with other therapeutic or investigational agents has been shown. Using in vitro models, enhancement of the inhibition of HCC cell proliferation with the combination of 5-fluorouracil and VK2 was shown [162], while cell-cycle arrest and apoptosis was demonstrated with sorafenib/VK2 [163]. A recent study in a clinic involving 44 HCC patients further demonstrated the efficacy and safety of VK2 and sorafenib combination treatment, where significantly higher objective response rates and extended median time of progression-free survival were observed in the combination treatment group compared to that of sorafenib alone [164].

In a prospective study of over 100,000 US adults, with a mean follow-up of over 8 years, dietary intake of VK1, but not VK2, appeared to confer a lower risk of pancreatic cancer [165]. In addition, Duan et al. studied the effect of VK2 on human bladder cancer cells, noting the induction of mitochondria-related cell apoptosis via ROS and JNK/p38 MAPK pathways [166] and promoted AMPK-dependent autophagic cell death via PI3K/AKT/HIF-1α-mediated glycolysis [167].

## 4. Conclusions

In summary, there is a growing body of evidence that VK2 could play an important pharmacological role in treating multiple disease conditions, including osteoporosis, osteoarthritis and rheumatoid arthritis, cardiovascular diseases, chronic kidney diseases, diabetes, neurodegenerative disorders and cancers. However, results from some clinical studies are still inconsistent. More in-depth investigations in VK2 research, including of metabolic processes, validation of biomarkers for VK2 status, dose–response and bioavailability, safety data, etc.) are still warranted. The establishment of a Recommended Daily Intake for VK2 is also needed, as this could have an important impact in improving global health.

## Data Availability

No new data were created or analyzed in this study. Data sharing is not applicable to this article.
